# Occupational Radiation Risk Stratification and Protection in Fluoroscopy-Guided Surgeons and Interventionalists: A Multispecialty Structured Narrative Review

**DOI:** 10.3390/jpm16010050

**Published:** 2026-01-13

**Authors:** Nana Kwadwo Okraku-Yirenkyi, Sri Snehita Reddy Bonthu, Hanisha Bhakta, Oluwatoyin O. Duyile, Michael Bernas

**Affiliations:** Anne Burnett School of Medicine, Texas Christian University, Fort Worth, TX 76104, USA; n.k.okraku@tcu.edu (N.K.O.-Y.); snehita.bonthu@tcu.edu (S.S.R.B.); hanisha.h.bhakta@tcu.edu (H.B.); o.duyile@tcu.edu (O.O.D.)

**Keywords:** occupational radiation exposure, fluoroscopy-guided procedures, shielding effectiveness, lead aprons, thyroid collars, lead glasses, cancer risk

## Abstract

**Background/Objectives:** Fluoroscopy-guided procedures are widely used across surgical and interventional specialties but expose operators to ionizing radiation with associated stochastic and deterministic effects. The aim is to characterize occupational radiation exposure, evaluate the effectiveness of shielding strategies, assess long-term cancer risks, and identify compliance patterns. **Methods:** This structured narrative review summarizes evidence on operator dose, shielding effectiveness, compliance with protective practices, and long-term cancer risk. A search of PubMed, Scopus, Embase, and Web of Science (limited to January 2000–March 2024) identified 62 records; 27 full texts were reviewed, and 16 studies met the inclusion criteria. **Results:** Across studies, unshielded chest exposure averaged 0.08–0.11 mSv per procedure, and eye exposure averaged 0.04–0.05 mSv. Lead aprons reduced exposure by about 90% at 0.25 mm and 99% at 0.5 mm, thyroid collars reduced neck dose by 60–70%, and lead glasses reduced ocular dose 2.5–4.5-fold. Compliance with aprons and thyroid collars was high, whereas lead glasses and lower-body shielding were inconsistently used. Limited epidemiologic data suggested a higher cancer burden in exposed surgeons, and Biologic Effects of Ionizing Radiation (BEIR) VII–based modeling projected increased lifetime risks of solid cancers in chronically exposed operators. **Conclusions:** Protective equipment substantially reduces operator dose, but exposure variability and inconsistent shielding practices persist and justify standardized monitoring, stronger enforcement of radiation safety protocols, and longitudinal studies.

## 1. Introduction

Fluoroscopy-guided procedures are increasingly integral to modern surgical practice, particularly in specialties such as orthopedic and trauma surgery, interventional cardiology, interventional radiology, vascular surgery, and cardiothoracic surgery. These techniques offer precision and minimally invasive approaches but also expose operators to ionizing radiation, presenting a persistent occupational hazard. A recent publication by Smith-Bindman et al. has highlighted the risk to physicians from work-related exposure to radiation in some specialties utilizing medical imaging [1].

Ionizing radiation is classified as a Group I human carcinogen by the International Agency for Research on Cancer [2]. International guidelines from organizations such as the International Commission on Radiological Protection (ICRP) recommend dose limits of 20 mSv per year with limits on equivalent dose to the lens of the eye of 20 mSv and of 500 mSv to the skin, hands and feet [3,4]. Exposure to this dose is associated with an additional lifetime risk of fatal cancer of 1 in 1000 [5]. Further guidelines emphasize ALARA (As Low As Reasonably Achievable) principles involving time, distance, and shielding to reduce exposure [3,4].

Radiation exposure can lead to stochastic effects, such as genetic aberrations and cancer that can occur randomly, and deterministic effects, such as hair loss and skin changes that occur when exposure exceeds a specified limit [6]. Beyond immediate exposure, long-term health risks, including cellular damage, cataracts, and malignancies such as skin cancer, breast cancer, thyroid cancer, and leukemia, have been documented [5,6,7]. Modeling studies, particularly those leveraging Biological Effects of Ionizing Radiation (BEIR) VII risk estimates, project elevated lifetime attributable risks (LAR)—for example, excess incidence of solid cancers and associated mortality for long-term occupational exposure [8]. Despite this, radiation safety training and systematic compliance remain inconsistent. Surveys highlight widespread gaps in education and monitoring, and adherence to protective practices such as dosimeter or shielding use, is frequently suboptimal [9,10].

Operators across specialties experience highly variable exposure patterns because procedure type, anatomic positioning, equipment configuration, and workflow differ substantially between fields such as orthopedics, interventional cardiology, and vascular surgery. These differences create distinct exposure profiles and long-term risk trajectories for different operator groups, highlighting the importance of risk stratification in evaluating occupational hazards. Understanding these specialty-specific patterns is therefore essential for ensuring that protective strategies and monitoring practices effectively address variations in operator risk. Because exposure profiles vary substantially across specialties, procedures, and operator roles, occupational radiation risk is inherently heterogeneous. Understanding and incorporating this variability aligns with personalized medicine principles by enabling risk-stratified protective strategies tailored to operator-specific exposure patterns. Primary specialties that utilize fluoroscopy include cardiology, orthopedics, gastroenterology, urology, vascular surgery, gynecology, and pain medicine. In fluoroscopy, X-rays constitute the primary form of ionizing radiation with both bremsstrahlung radiation and characteristic X-rays [11]. The energy range for X-ray photons used in fluoroscopy is typically between 40 and 125 kilovolts peak (kVp).

Within this context, we conducted a structured narrative review encompassing a range of surgical and interventional specialties that utilize fluoroscopy, aiming to characterize occupational radiation exposure, evaluate the effectiveness of shielding strategies, assess long-term cancer risks, and identify compliance patterns.

## 2. Materials and Methods

This structured narrative review followed guidelines from Sukhera [12] and utilized a comprehensive literature search independently conducted between March and May 2025 using PubMed, Scopus, Embase, and Web of Science. The Boolean search strategy was used to identify relevant literature. The search string used in the electronic databases was: “radiation protection” OR “lead vest” OR “lead apron” OR “thyroid shield” OR “radiation shielding” AND “occupational exposure” OR “operator dose” OR “fluoroscopy” OR “interventional procedures” OR “surgeon” OR “interventional cardiology” OR “orthopedic surgery” OR “vascular surgery” OR “interventional radiology.” This initial search strategy resulted in over 10,000 results that were a logistical challenge. Therefore, smaller, targeted Boolean variants (accomplished by reducing the number of search terms) were generated iteratively to improve search specificity. Filters were also applied to limit results to human studies, English-language publications, and peer-reviewed articles between 2000 and 2024. Inclusion and exclusion criteria (Table 1) were also utilized (see below). Additionally, reference lists of highly relevant papers were manually screened to identify supplementary eligible studies.

### 2.1. Article Selection

All studies identified in the search were eligible for review. The initial search yielded 62 articles after duplicate removal. Two independent authors screened titles and abstracts for relevance to occupational radiation exposure and protection strategies and retrieved 27 articles for full-text review. After applying the inclusion and exclusion criteria detailed in Table 1 at the full-text stage, 16 articles were included in the final synthesis. These studies spanned multiple specialties, procedural settings, and research methodologies, offering a broad evidence base to explore trends and best practices in occupational radiation safety during fluoroscopy-guided interventions. Although there was high methodological heterogeneity of the identified studies, inclusion and exclusion criteria is tight enough to capture relevant data.

### 2.2. Data Extraction

A detailed, pre-designed spreadsheet extraction template was used to collect data systematically across 25 structured fields: title, year, whether it was primary research, study design, sample size, country or region, surgical or interventional specialty, study duration or follow-up period, whether radiation exposure was measured or estimated, use of lead aprons or other shielding, consistency of lead protection use, pre- versus post-lead vest or shielding data, cancer type studied and its incidence or prevalence if applicable, whether a comparison group was included, reported risk ratios, odds ratios, or hazard ratios if applicable, confounding variables considered, lifestyle or occupational exposures assessed, statistical significance reported, whether longitudinal effects were studied, any policy or protocol recommendations, compliance with protection protocols, innovative methodologies used, inclusion of a pre-shielding or pre-vest historical baseline, and the role of that historical baseline in the paper.

Manual extraction was first performed by a single reviewer with secondary reviews from additional authors with cross-checking to ensure consistency and accuracy. Emphasis was placed on numeric values (e.g., radiation dose in mSv, percentage dose reductions, fold-change effects, risk multipliers) and statistically significant results. When numeric data were not explicitly reported, dose-related information was extracted from descriptive statements when possible; otherwise, values were marked as Not Reported.

### 2.3. Data Analysis

Extracted data were analyzed to calculate overall and subgroup-level summary statistics–including means, medians, ranges–for operator doses, shielding-associated reductions, and compliance rates. Trends were compared across surgical and interventional specialties including orthopedics, trauma, interventional cardiology, interventional radiology, vascular surgery, and cardiothoracic surgery. Procedural factors associated with higher or lower exposure, such as fluoroscopy time, beam angle, C-arm positioning, and the use of real-time dosimetry, were identified. Common methodological strengths, weaknesses, and gaps in the existing literature were synthesized and the consistency and robustness of reported statistical significance across diverse study types were assessed.

Due to methodological heterogeneity, no formal meta-analysis or pooled effect size calculations were performed. Instead, the review presents a qualitative and quantitative synthesis of available data, highlighting both generalizable insights and areas requiring further specialty-specific investigation.

## 3. Results

**Figure 1:**
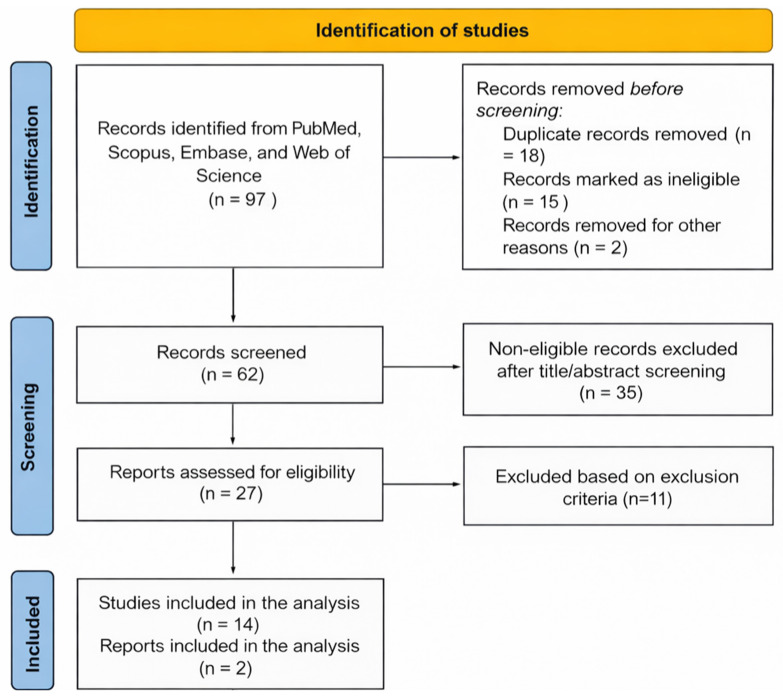
A PRISMA-style flow diagram of the narrative review showing the identification, screening, eligibility assessment, and inclusion of studies for final inclusion and anlaysis.

A total of 16 studies were included in this review (Table 2). The narrative review search flow chart is depicted in Figure 1. The studies spanned a variety of surgical and interventional specialties, including orthopedic surgery [13,14,15,16,17,18], trauma surgery [14], interventional radiology [19], cardiology [20,21], and vascular surgery [22,23,24,25,26,27,28]. Publication dates ranged from 2000 to 2024, with the majority concentrated between 2010 and 2024. Eleven studies (69%) were primary research studies, either prospective or retrospective, while the remaining five were high-quality review or modeling articles (Table 2). Data originated from the United States, Italy, Japan, Ireland, South Korea, and multinational datasets, including large-scale analyses such as atomic bomb survivor follow-ups and BEIR VII risk models.

### 3.1. Sample Characteristics

Sample sizes ranged from large-scale epidemiological datasets (*n* ≈ 120,000) [13] to small intraoperative measurement cohorts (*n* = 60 procedures) [14]. Operator-specific exposure data were reported across all studies. Vascular (7), orthopedic (6) and trauma (5) surgery represented the largest specialty groups, followed by cardiology (2), interventional radiology (1). Cardiothoracic surgery was minimally represented, largely through modeled rather than directly measured exposure data. Follow-up durations ranged from six weeks to over 50 years for studies incorporating long-term cancer risk projections, with several extrapolating annual exposure from short-term dosimetry findings.

### 3.2. Radiation Exposure and Shielding Effectiveness

Intraoperative exposures were consistently reported. Without protective gear, chest-level doses averaged 0.08–0.11 mSv per procedure, and eye-level doses averaged 0.04–0.05 mSv per procedure. High-volume trauma surgeons demonstrated knee-thigh doses up to 2.7 mSv annually. When shielding was employed, attenuation was substantial: 0.25 mm lead aprons reduced exposure by ~90%, while 0.5 mm aprons achieved a reduction of ~99%. Lead glasses decreased ocular doses 2.5–4.5-fold, and thyroid collars reduced neck-level exposure by approximately 60–70%. Real-time dosimetry and phantom modeling confirmed shielding efficacy, with external-to-internal dose reductions up to 91.1%.

### 3.3. Cancer Incidence and Modeled Risk

Direct evidence of cancer incidence was limited, largely modeled or extrapolated, but notable. One Italian orthopedic study reported a 29% cancer incidence among exposed surgeons compared with 4% in non-exposed controls [13,16]. U.S. data showed a 2.9-fold higher breast cancer rate among female orthopedic surgeons, while cardiology studies indicated a median latency of ~22 years for head and neck tumors [13]. Modeled lifetime attributable risks (LAR) per million exposed individuals, based on BEIR VII data, included colon cancer at 424 (≈1 in 236), lung cancer at 447 (≈1 in 224), all solid cancers for male surgeons at 2079 (≈1 in 48), and cancer-related mortality across all sites at 1309 (≈1 in 76) [22]. These risk estimates were based on BEIR VII reports and extrapolated occupational exposures, assuming careers spanning ages 18–65 at consistent annual doses. These extrapolated results may not imply direct causality.

### 3.4. Comparison Groups and Risk Ratios

Several studies incorporated internal or external comparison groups, such as non-exposed staff or population baselines [13,15,21,22]. Reported effect sizes included a relative risk (RR) of 1.6 for solid cancers per 1 Sv exposure (atomic bomb survivor data), a hazard ratio (HR) of 4.04 for high-procedure cardiologists compared to low-procedure peers, and an RR of 3.2 (*p* < 0.005) for posterior lens opacities (cataracts).

### 3.5. Protection Protocols and Compliance

Lead aprons and thyroid collars demonstrated high compliance rates (90–99%) [15], while lead glasses showed inconsistent use [15,22,23,24], often due to interference with surgical loupes [22]. Seated procedures frequently showed unshielded gaps at the apron skirt edge, raising gonadal dose exposure. Several studies highlighted routine equipment integrity checks, real-time dosimeter use, and institutional enforcement of protective protocols as key areas for improvement.

### 3.6. Innovative Methodologies

Methodologies included real-time electronic personal dosimeters (EPD) and thermoluminescent dosimeters (TLD) mapped across body regions, phantom models simulating scatter radiation at variable distances and angles, and procedure-specific modeling comparing exposures across endovascular aneurysm repair (EVAR), angioplasty, and trauma cases.

### 3.7. Summary Statistics

Summary statistics are presented in Table 3. Across studies, the mean sample size was approximately 4200 (median 1200; range 60–120,000). Mean chest-level dose was ~0.10 mSv per procedure, and mean eye-level dose without lead was ~0.05 mSv per procedure. Gonadal exposure for busy trauma surgeons averaged ~2.7 mSv annually. The estimated relative risk for solid cancer per Sv was consistently reported at 1.6 (SD 0.1).

### 3.8. Key Trends and Insights

Key findings included: (1) Across specialties, shielding via lead aprons, thyroid collars, and glasses provided substantial dose reductions by 60–99% depending on body region and material thickness; (2) compliance gaps persisted, particularly regarding lead glasses usage and coverage of apron skirt edges; (3) phantom and modeled data closely mirrored measured exposure patterns via dose dosimetry but highlighted variability across procedures and operator roles; (4) direct specialty-specific cancer data remain scarce, with most conclusions extrapolated from surrogate dose reductions and modeled lifetime risks; and (5) practices and safety protocols differed markedly across regions, with international comparisons often referencing U.S. and European standards.

## 4. Discussion

This structured narrative review across 16 studies provides valuable insights into occupational radiation exposure among surgical and interventional proceduralists. While fluoroscopy-guided techniques continue to enhance minimally invasive care, cumulative ionizing radiation exposure remains an underrecognized occupational hazard, especially due to inconsistent protective practices and insufficient long-term outcome data.

Across included studies, operator doses reported were unshielded ~0.08–0.11 mSv/procedure at the chest and ~0.04–0.05 mSv/procedure at the eye. Lead aprons reducing exposure by ~90–99% and lead glasses reducing eye dose ~2.5–4.5×) can be benchmarked against current occupational limits to judge whether fluoroscopy-guided practice is typically “acceptable.” Under contemporary international guidance, the occupational effective-dose limit is 20 mSv/year averaged over 5 years (≤50 mSv in any single year), the lens limit is 20 mSv/year averaged over 5 years (≤50 mSv in any single year), and skin/extremities are limited at 500 mSv/year [2,29,30]. U.S. regulations permit a whole-body effective dose of 50 mSv/year, 150 mSv/year to the lens, and 500 mSv/year to the skin/extremities [31]. Interpreted against these thresholds, the per-procedure doses summarized here imply that routine practice with consistent shielding is generally compatible with remaining below annual effective-dose limits, while the lens and (in specific workflows) the hands are the most plausible limit-driving components. However, meeting dose limits should not be conflated with being “risk-averse”; limits are regulatory/occupational compliance thresholds, whereas stochastic effects such as cancer are probabilistic and are reduced by dose minimization rather than eliminated below any particular cutoff. Therefore, “acceptable risk” in fluoroscopy-guided practice is best interpreted as compliance plus optimization (ALARA), emphasizing consistent PPE, geometry optimization, and dosimetry strategies that specifically capture high-risk patterns of eye and extremity exposure in high-volume operators.

### 4.1. Shielding Efficacy and Practice Patterns

Our synthesis reaffirms that protective gear ranging from 0.25 mm to 0.5 mm lead-equivalent aprons, thyroid collars, and lead glasses provides substantial dose reduction (60–99%) across body regions. This aligns with existing recommendations that employ a combination of time, distance, and shielding (ALARA principles) to mitigate exposure risk. Additionally, newer comparative work indicates that traditional lead aprons often outperform lightweight alternatives, underscoring the importance of material integrity and equivalent thickness. These aprons should be considered for wider use.

Despite strong evidence supporting personal protective equipment (PPE), compliance gaps remain. While there is adherence to the usage of thyroid collars and aprons, lead glasses, crucial for eye protection, are underutilized, often due to interference with surgical optics (e.g., loupes). This supports broader findings that surgical trainees and attendings often express inadequate radiation safety education and concern over long-term risk, particularly regarding fertility and cancer. This area is a current actionable recommendation.

The variability observed across specialties, procedural roles, and exposed body regions highlights that occupational radiation risk is not uniform. Orthopedic trauma surgeons, interventional cardiologists, and other high-fluoroscopy practitioners demonstrate distinct exposure patterns, dose magnitudes, and long-term risk profiles. These differences underscore the need for risk-stratified approaches to occupational protection, in which monitoring practices, shielding selection, and institutional protocols are adapted to the specific exposure characteristics of different operator groups. Such stratification reflects an individualized framework consistent with modern precision-oriented occupational health. These specialty-specific exposure differences reinforce a precision-oriented approach to occupational health in which monitoring practices and protective strategies are tailored to the operator’s individualized risk profile, aligning this work with core principles of personalized medicine for protection of physicians using fluoroscopy in their practices.

### 4.2. Risk Estimation and Long-Term Health Outcomes

Direct epidemiological data on cancer incidence among operators are limited but concerning, nonetheless. One study reported markedly higher cancer rates in exposed orthopedic surgeons than controls, and elevated breast cancer rates among female surgeons were highlighted in U.S. data. Most risk assessments, however, rely on modeling using BEIR VII data to estimate lifetime attributable risk of cancers and mortality (e.g., ~1 in 48 for solid cancers; ~1 in 76 for cancer-related mortality). These estimates reflect a need for comprehensive occupational registries and longitudinal research.

Risk estimates from atomic bomb survivors yielding an RR of 1.6 per 1 Sv may not directly apply to proceduralists, whose exposures are typically lower and chronic. Nevertheless, findings such as a hazard ratio of 4.04 for high-volume interventional cardiologists and a relative risk of approximately 3.2 for cataracts indicate that even procedural-level exposures may carry significant health risks, though study heterogeneity limits meta-analysis and strong causal inference.

### 4.3. Implications for Practice and Future Research

Institutional commitment to comprehensive safety measures is imperative to better protect operators. Strategies such as routine integrity checks for protective gear, standardized compliance monitoring with dosimetry, and appointing a Radiation Safety Officer (RSO) to oversee adherence to ALARA principles have proven effective in other settings. Education tailored to proceduralists, potentially through advanced tools such as virtual reality (VR)-based radiation field visualization, could further improve compliance [32]. The importance of local diagnostic reference levels (DRLs) in the context of risk stratification and optimization of radiation protection for operators is also emphasized by recent studies indicating that taking into account local procedural and equipment conditions allows for a more accurate assessment of exposure and more effective implementation of ALARA principles [33]. Additionally, procedural modifications such as maintaining modest distance from scatter sources, beam collimation, pulsed fluoroscopy, and integrating ceiling-mounted shielding in hybrid OR setups can cumulatively reduce exposure.

Future research should prioritize prospective studies that track cumulative exposure and long-term outcomes such as cancer and other stochastic effects. Standardized reporting is needed to enable cross-study comparisons and meta-analysis. More studies will also reduce the heterogeneity of results and should allow subgroup analysis. Incorporating real-time dosimetry, phantom modeling, and registry-linked data can improve risk estimates, while studies testing and evaluating new shielding technologies, training strategies, and workflow changes may identify effective approaches to strengthen radiation safety practices.

### 4.4. Limitations

This structured narrative review has several limitations. Considerable heterogeneity was present in study designs, outcome measures, and reporting methods across the included papers. Longitudinal cancer outcome data were limited, as most studies focused on immediate or modeled radiation exposure rather than long-term health effects. Some specialties were underrepresented compared with others—for example, cardiothoracic surgery had fewer studies relative to orthopedics or interventional cardiology to generate appropriate strong data. In addition, adherence to protective equipment protocols and the rigor of monitoring practices varied substantially across institutions and operator groups. In addition, cancer risk models based on BEIR VII data may not be adequate for chronic, low-dose occupational exposure characteristic of fluoroscopy operators. Nevertheless, the aggregated findings provide valuable insights into the current state of occupational radiation safety practices in fluoroscopy-guided procedural specialties.

## 5. Conclusions

Operators across surgical and interventional specialties face considerable exposure to ionizing radiation during fluoroscopically guided procedures. Although protective measures achieve significant dose reductions, gaps in compliance, knowledge, and protocol implementation persist. Evidence linking exposure to long-term health outcomes remains limited, underscoring the need for standardized monitoring, rigorous safety practices, and well-designed longitudinal studies to guide future recommendations. The marked variability in exposure across specialties, procedural roles, and exposed body regions indicates that occupational risk is not uniform. Integrating risk-stratified monitoring and protective protocols may better address the distinct exposure patterns encountered by different operator groups and align with emerging precision-oriented approaches to occupational health. Anchoring these strategies within a personalized medicine framework underscores the need to tailor occupational protections to operator-specific exposure patterns, ensuring safety practices that reflect the individualized risk profiles identified in this review.

## Figures and Tables

**Table 1:** Inclusion and exclusion criteria for article selection.

Inclusion Criteria	
1. Topic Relevance	Addressed occupational radiation exposure, operator safety, or the effectiveness of protective measures (e.g., lead aprons, thyroid collars, lead glasses, leg shields, table skirts, ceiling-mounted barriers) during fluoroscopy-guided procedures.
2. Specialty Scope	Included any surgical or interventional specialty using fluoroscopy, such as orthopedics, trauma surgery, interventional cardiology, interventional radiology, vascular surgery, or cardiothoracic surgery.
3. Outcome Measures	Reported quantitative data on measured or modeled operator radiation dose, shielding-related dose reductions, procedural factors influencing exposure, or compliance with safety protocols.
4. Study Types	Peer-reviewed primary research (prospective, retrospective, or experimental), cohort studies, phantom or cadaver simulations, and high-quality narrative reviews with quantitative findings.
5. Language	English
6. Publication Time Frame	January 2000 to March 2024, ensuring relevance to modern imaging systems and protective technologies.
1. Articles that focused solely on patient radiation exposure without reporting operator outcomes	
2. Articles that provided no quantitative metrics or measurable findings related to operator dose or protection effectiveness	
3. Non-peer-reviewed studies (e.g., editorials, opinion pieces, conference abstracts without full texts).	
4. Studies that investigated non-fluoroscopic fields (e.g., external beam radiotherapy, nuclear medicine not using fluoroscopy, dental radiography).	

**Table 2:** Characteristics of the included studies. Reference numbers correspond to the first citation of each study, as established in this table and used consistently throughout the manuscript.

Author, Year Published	Study Type	Specialty	Summary Results	Reference
Hayda et al., 2018	Review article	Orthopaedic surgery—trauma, spine, general ortho; also referenced interventional cardiology, radiology for comparison	This article discusses the health risks associated with radiation exposure in orthopedic surgery, emphasizing the importance of protective measures to mitigate cancer risks. While focused on orthopedic surgeons, the findings are relevant to other surgical specialties that utilize fluoroscopy.	[13]
Kim et al., 2017	Prospective measurement study	Vascular surgery	Although yearly radiation hazards for vascular surgeons and scrub nurses are still within safety guidelines, protection principles can never be too stringent when aiming to minimize the cumulative harmful effects.	[22]
Hurley et al., 2022	Prospective observational study	Trauma and Orthopaedic surgery	PPE currently used by surgeons in orthopaedic trauma theatre adequately reduces radiation exposure to below recommended levels. Normative data per trauma case show specific an- atomical areas of higher exposure, which may benefit from enhanced radiation protection. EPDs can be used to assess real-time radiation exposure in orthopaedic surgery.	[14]
Wan et al., 2021	Narrative review	Orthopedic surgery—trauma, spine, foot and ankle, sports medicine, general orthopedics	Fluoroscopy brings radiation hazard to the physicians and other staffs in operating theatre. Risk of leukaemia, risk to eyes, thyroid, skin, breast and fetes cannot be underestimated. Physician should properly use protective equipment including lead aprons, thyroid shields, radiation protection goggles and gloves, and dosimeter, to protect one-self from the invisible radiation hazard. The medical field should observe principles of justification and optimization, to reduce the radiation hazard to ALARA. Proper understanding of radiation risk will encourage doctors to use proper protection gear, and to clear unnecessary worries. Formal training on radiation safety and safe fluoroscopy use should also be encouraged and adopted.	[15]
Peri Ho et al., 2007	Prospective observational study	Vascular surgery—endovascular procedures	With current radiation protection practice, the radiation absorbed by vascular surgeons with a high endovascular workload did not exceed the safety limits recommended by ICRP. Variations in practice, however, can result in significant discrepancy of radiation absorption between surgeons.	[23]
Agarwal 2011	Narrative review	Orthopedic surgery—general, trauma, spine, foot and ankle	Orthopedic surgeons should use protective measures, which include lead aprons, thyroid shields, and, if at all possible, lead goggles. Techniques described to minimize direct and scatter exposure to the hands, eyes, and neck should be used. Measures to protect the patient should also be used intraoperatively, if feasible, such as shielding of the gonads. This is even more important as the awareness of patient radiation exposure increases.	[16]
Sritharan et al., 2024	Cross-sectional online survey	Vascular surgery	This survey highlights significant and concerning deficiencies in knowledge, access to personal radiation protection and failures in monitoring individual exposure to ionising radiation amongst the UK vascular surgical workforce.	[24]
Massey et al., 2022	Retrospective case-control study	Orthopedic surgery—general, trauma, C-arm fluoroscopy-assisted procedures	Institution of a PLP increased the compliance and exposure readings of radiation dosimeter badges for orthopedic surgery residents, whereas the actual monthly fluoroscopy time did not change. Teaching hospitals should consider implementing a PLP to more accurately monitor exposure.	[17]
Bhinder et al., 2023	Prospective quality improvement study	Vascular surgery—endovascular, hybrid OR, angiography	Safety policies in place at vascular residency and fellowship programs were inadequate in securing the safety of their trainees. Interventions such as inventorying and ensuring availability of safety equipment, hands-on instruction to complement traditional didactics, lowering the default frame rates, and converting to real-time dosimetry to be effective measures for reducing radiation exposure.	[25]
Kirkwood et al., 2015	Prospective observational study	Vascular surgery—complex endovascular interventions	Surgeon radiation dose during FGIs depends on case type, operator position, and table skirt use but not on the level of fellow training. On the basis of these data, the primary operator could perform approximately 12 FEVARs/wk and have an annual dose <10 mSv, which would not exceed lifetime occupational dose limits during a 35-year career. With practical case loads, operator doses are relatively low and unlikely to exceed occupational limits.	[26]
Harstall et al., 2005	Prospective case-control study	Orthopaedic spine surgery	While performing percutaneous vertebroplasty, the surgeon is exposed to a significant amount of radiation. Proper surgical technique and shielding devices to decrease potentially high morbidity are mandatory. Training in radiation protection should be an integral part of the education for all surgeons using minimally invasive radiologic-guided interventional techniques.	[18]
Haqqani et al., 2012	Prospective controlled study	Vascular surgery—endovascular procedures, digital subtraction angiography	Varying imaging techniques results in different radiation exposure to members of an endovascular surgery team. Knowledge of the variable intensity of radiation exposure may allow modification of the technique to minimize radiation exposure to the team while providing suitable imaging.	[27]
Dorey et al., 2019	Controlled laboratory experiment	Interventional radiology	If the clinician’s gaze is directed towards the main scattering source, a potential exists for reducing eye lens dose compared with fixed location computer monitors. Introduction of lead lined smart glasses into interventional radiology may lead to improvements in patient care, reducing the need for the clinician to look away from the patient to observe a radiographic image.	[19]
Kastrati et al., 2016	Retrospective Observational Study	Cardiology—coronary angiography and angioplasty	A new x-ray technology with image noise reduction algorithm provides a substantial reduction in radiation exposure without the need to prolong the procedure or fluoroscopy time.	[20]
Modarai 2022	Expert commentary	Vascular surgery—endovascular procedures	Vascular operators are at significant occupational risk from cumulative ionizing radiation exposure, with potential long-term health consequences. Consistent use of protective equipment, adherence to radiation-minimizing techniques, and increased awareness and education are essential to reduce exposure and improve operator safety.	[28]
Picano et al., 2024	Comprehensive review	Cardiology—diagnostic and interventional radiology, nuclear cardiology, electrophysiology	Review updates estimated societal health burden from diagnostic radiology and nuclear medicine, showing that rising population radiation exposure is associated with higher-than-previously-recognized risks of both cancer and cardiovascular disease. Emerging epidemiological evidence refutes the assumption that low-dose radiation is biologically negligible, and combined cancer and cardiovascular risks are now understood to impose a substantially greater public health impact than earlier estimates suggested.	[21]

**Table 3:** Summary statistics for operator exposure metrics across included studies.

Metric	Mean	Median	Range	Std. Dev.
Sample Size (where reported)	~4200	~1200	60–120,000	High spread
Chest-level dose (mSv/procedure)	~0.10	0.09	0.08–0.11	0.01
Eye-level dose (mSv/procedure, no lead)	~0.05	0.045	0.04–0.07	0.01
Gonadal dose (mSv/year, busy trauma surgeons)	~2.7	2.5	2.4–3.8	0.5
Estimated risk ratios (solid cancer, per Sv)	1.6	1.6	1.5–1.7	0.1

## Data Availability

All data discussed in this review are drawn from previously published studies and are available within the cited literature.

## Abbreviations

The following abbreviations are used in this manuscript:

BEIR	Biological Effects of Ionizing Radiation
ALARA	As Low As Reasonably Achievable
LAR	Lifetime Attributable Risks
ICRP	International Commission on Radiological Protection

## References

[1] Smith-Bindman R., Chu P.W., Firdaus H.A., Stewart C., Malekhedayat M., Alber S., Bolch W.E., Mahendra M., de González A.B., Miglioretti D.L. (2025). Projected Lifetime Cancer Risks from Current Computed Tomography Imaging. JAMA Intern. Med..

[2] El Ghissassi F., Baan R., Straif K., Grosse Y., Secretan B., Bouvard V., Benbrahim-Tallaa L., Guha N., Freeman C., Galichet L. (2009). A review of human carcinogens--part D: Radiation. Lancet Oncol..

[3] López P.O., Dauer L., Loose R., Martin C., Miller D., Vañó E., Doruff M., Padovani R., Massera G., Yoder C. (2018). on behalf of the ICRP. ICRP Publication 139: Occupational radiological protection in interventional procedures. Ann. ICRP.

[4] Stewart F.A., Akleyev A.V., Hauer-Jensen M., Hendry J.H., Kleiman N.J., Macvittie T.J., Aleman B.M., Edgar A.B., Mabuchi K., Muirhead C.R. (2012). ICRP publication 118: ICRP statement on tissue reactions and early and late effects of radiation in normal tissues and organs--threshold doses for tissue reactions in a radiation protection context. Ann. ICRP.

[5] Matityahu A., Duffy R.K., Goldhahn S., Joeris A., Richter P.H., Gebhard F. (2017). The Great Unknown-A systematic literature review about risk associated with intraoperative imaging during orthopaedic surgeries. Injury.

[6] Hamid M.A., Younis Z., Raza A., Tauseef A., Khan M.A.H., Mir S., Abdulsattar S., Rashid N. (2025). Radiation in the Orthopedic Operating Room: What We Know, What We Do, and What Needs Attention. Cureus.

[7] Calgary Orthopaedic Resident Research Group (2024). Quantification of Radiation Exposure in Canadian Orthopaedic Surgery Residents. JBJS Open Access.

[8] National Research Council (US) Committee on Health Effects of Exposure to Low Levels of Ionizing Radiations (BEIR VII) (1998). Health Effects of Exposure to Low Levels of Ionizing Radiations: Time for Reassessment?.

[9] Aljohmani L., Gaffney A., Kelly L., O’Sullivan L.-A., Leyva E., O’Connor M., McCavana J., Heffernan E., Quinlan C., Dolan R. (2025). Knowledge gaps and radiation exposure concerns: Time for a revamp of radiation training structures for trainee surgeons. Surgeon.

[10] Kang S., Cha E.S., Bang Y.J., Na T.W., Lee D., Song S.Y., Lee W.J. (2020). Radiation exposure and fluoroscopically-guided interventional procedures among orthopedic surgeons in South Korea. J. Occup. Med. Toxicol..

[11] Rivera K., Ahn S.S. (2025). Radiation Physics and Safety in Fluoroscopy: A Clinician’s Guide to Principles and Practice. Cureus.

[12] Sukhera J. (2022). Narrative Reviews: Flexible, Rigorous, and Practical. J. Grad. Med. Educ..

[13] Hayda R.A., Hsu R.Y., DePasse J.M., Gil J.A. (2018). Radiation Exposure and Health Risks for Orthopaedic Surgeons. J. Am. Acad. Orthop. Surg..

[14] Hurley R.J., McCabe F.J., Turley L., Maguire D., Lucey J., Hurson C.J. (2022). Whole-body radiation exposure in Trauma and Orthopaedic surgery. Bone Jt. Open.

[15] Wan R.C.W., Chau W.W., Tso C.Y., Tang N., Chow S.K., Cheung W.-H., Wong R.M. (2021). Occupational hazard of fluoroscopy: An invisible threat to orthopaedic surgeons. J. Orthop. Trauma. Rehabilit..

[16] Agarwal A. (2011). Radiation Risk in Orthopedic Surgery: Ways to Protect Yourself and the Patient. Oper. Tech. Sports Med..

[17] Massey P.A., Myers M.E., Guedry R.D., Lowery M.T., Perry K.J., Barton R.S. (2022). Improved Radiation Exposure Monitoring of Orthopaedic Residents After Institution of a Personalized Lead Protocol. JBJS Open Access.

[18] Harstall R., Heini P.F., Mini R.L., Orler R. (2005). Radiation exposure to the surgeon during fluoroscopically assisted percutaneous vertebroplasty: A prospective study. Spine.

[19] Dorey S., Gray L., Tootell A., Higgins R., Al-Islam S., Baxter H., Dixon P., Hogg P. (2019). Radiation protection value to the operator from augmented reality smart glasses in interventional fluoroscopy procedures using phantoms. Radiography.

[20] Kastrati M., Langenbrink L., Piatkowski M., Michaelsen J., Reimann D., Hoffmann R. (2016). Reducing Radiation Dose in Coronary Angiography and Angioplasty Using Image Noise Reduction Technology. Am. J. Cardiol..

[21] Picano E., Vañó E. (2024). Updated Estimates of Radiation Risk for Cancer and Cardiovascular Disease: Implications for Cardiology Practice. J. Clin. Med..

[22] Kim J.B., Lee J., Park K. (2017). Radiation hazards to vascular surgeon and scrub nurse in mobile fluoroscopy equipped hybrid vascular room. Ann. Surg. Treat. Res..

[23] Ho P., Cheng S.W.K., Wu P.M., Ting A.C., Poon J.T., Cheng C.K., Mok J.H., Tsang M. (2007). Ionizing radiation absorption of vascular surgeons during endovascular procedures. J. Vasc. Surg..

[24] Sritharan K., Sheikh Z., Saratzis A., Lakshminarayan R., Garnham A., Bowbrick G., Morgan R. (2024). Standards of radiation protection amongst UK vascular surgeons: A clinician’s perspective. J. Vasc. Soc. Great Br. Irel..

[25] Bhinder J., O’Brien-Irr M., Reilly B., Montross B., Khan S., Rivero M., Cherr G., Harris L. (2023). Understanding Radiation Exposure and Improving Safety for Vascular Surgery Trainees. J. Vasc. Surg..

[26] Kirkwood M.L., Guild J.B., Arbique G.M., Anderson J.A., Valentine R.J., Timaran C. (2015). Surgeon radiation dose during complex endovascular procedures. J. Vasc. Surg..

[27] Haqqani O.P., Agarwal P.K., Halin N.M., Iafrati M.D. (2012). Minimizing radiation exposure to the vascular surgeon. J. Vasc. Surg..

[28] Endovascular Today (2022). Radiation Exposure, Effects, and Safety Measures for Vascular Operators (with Bijan Modarai, PhD, FRCS).

[29] International Commission on Radiological Protection (2011). Statement on Tissue Reactions.

[30] International Atomic Energy Agency (2014). Radiation Protection and Safety of Radiation Sources: International Basic Safety Standards. IAEA Safety Standards Series No. GSR Part 3.

[31] U.S. Nuclear Regulatory Commission Standards for Protection Against Radiation. 10 CFR § 20.1201 Occupational Dose Limits for Adults. Electronic Code of Federal Regulations. https://unblock.federalregister.gov/.

[32] Unberath M., Fotouhi J., Hajek J., Maier A., Osgood G., Taylor R., Armand M., Navab N. (2018). Augmented reality-based feedback for technician-in-the-loop C-arm repositioning. Health Technol. Lett..

[33] Modlińska S., Rojek M., Bielówka M., Kufel J. (2024). Establishing Local Diagnostic Reference Levels for Head Computed Tomography Examinations. Biomedicines.

